# Examination of Genome-Wide Ortholog Variation in Clinical and Environmental Isolates of the Fungal Pathogen Aspergillus fumigatus

**DOI:** 10.1128/mbio.01519-22

**Published:** 2022-06-29

**Authors:** Maria Augusta C. Horta, Jacob L. Steenwyk, Matthew E. Mead, Luciano H. Braz dos Santos, Shu Zhao, John G. Gibbons, Marina Marcet-Houben, Toni Gabaldón, Antonis Rokas, Gustavo H. Goldman

**Affiliations:** a Faculdade de Ciências Farmacêuticas de Ribeirão Preto, Universidade de São Paulo, São Paulo, Brazil; b Department of Biological Sciences, Vanderbilt University, Nashville, Tennessee, USA; c Vanderbilt Evolutionary Studies Initiative, Vanderbilt University, Nashville, Tennessee, USA; d Brazilian Agricultural Research Corporation, Embrapa Agroenergy, Brasília, Brazil; e Molecular and Cellular Biology Graduate Program, University of Massachusetts, Amherst, Massachusetts, USA; f Department of Food Science, University of Massachusetts, Amherst, Massachusetts, USA; g Organismic and Evolutionary Biology Graduate Program, University of Massachusetts, Amherst, Massachusetts, USA; h Barcelona Supercomputing Centre, Barcelona, Spain; i Institute for Research in Biomedicine, The Barcelona Institute of Science and Technology, Barcelona, Spain; j Catalan Institution for Research and Advanced Studies, Barcelona, Spain; k Centro de Investigación Biomédica En Red de Enfermedades Infecciosas, Barcelona, Spain; Duke University Medical Center

**Keywords:** *Aspergillus fumigatus*, PanOrtho genome, clinical and environmental isolates, core and accessory genes

## Abstract

Aspergillus fumigatus is both an environmental saprobe and an opportunistic human fungal pathogen. Knowledge of genomic variation across A. fumigatus isolates is essential for understanding the evolution of pathogenicity, virulence, and resistance to antifungal drugs. Here, we investigated 206 A. fumigatus isolates (133 clinical and 73 environmental isolates), aiming to identify genes with variable presence across isolates and test whether this variation was related to the clinical or environmental origin of isolates. The PanOrtho genome of A. fumigatus consists of 13,085 ortholog groups, of which 7,773 (59.4%) are shared by all isolates (core groups) and 5,312 (40.6%) vary in their gene presence across isolates (accessory groups plus singletons). Despite differences in the distribution of orthologs across all isolates, no significant differences were observed among clinical versus environmental isolates when phylogeny was accounted for. Orthologs that differ in their distribution across isolates tend to occur at low frequency and/or be restricted to specific isolates; thus, the degree of genomic conservation between orthologs of A. fumigatus is high. These results suggest that differences in the distribution of orthologs within A. fumigatus cannot be associated with the clinical or environmental origin of isolates.

## INTRODUCTION

Aspergillus fumigatus is a soilborne and ubiquitously distributed filamentous fungus that recycles organic matter. A. fumigatus is also a major opportunistic human pathogen that causes life-threatening invasive pulmonary aspergillosis (IPA) in immunocompromised hosts. A. fumigatus is responsible for an estimated 3,000,000 cases of aspergillosis annually and more than 200,000 cases of IPA each year, reaching a mortality rate of up to 90% in the most susceptible populations. One reason for the prevalence of A. fumigatus as the main clinical etiological agent of aspergillosis is its cosmopolitan distribution and high prevalence in the environment, with the average human inhaling hundreds of airborne asexual spores (conidia) of A. fumigatus daily (1, 2, 62).

Genetic heterogeneity of A. fumigatus has been extensively described (3–10). A. fumigatus also exhibits phenotypic heterogeneity in virulence in animal models of IPA (5, 11) and in its drug resistance profiles to antifungals (12). Azoles are the first-line treatment against IPA, but the recent emergence of azole resistance in A. fumigatus has made treatment even more challenging (1). Resistance to azoles is an important concern, and global surveillance studies reveal that 3.2% of A. fumigatus isolates are resistant to one or more azoles, with large discrepancies between geographic regions (13). The azole target is the 14-α-sterol demethylase encoded by *cyp51A*, and the main mechanisms of resistance found in environmental and clinical isolates involve mutations in the *cyp51A* coding region and tandem duplications in its promoter region (1, 14). The genetic heterogeneity of the A. fumigatus population with regard to azole resistance has been described (10, 14, 15).

Several recent studies explored the genetic diversity across A. fumigatus isolates, with some of these studies aiming to explain how the observed genomic diversity contributes to variation in secondary metabolism, virulence, and antifungal drug resistance (6–8, 10, 16). Zhao and Gibbons (7) looked at gene copy number variation across A. fumigatus isolates and found gene presence-absence polymorphisms associated with virulence. Lofgren et al. (10) analyzed gene sequence and gene presence-absence variation to support the existence of three distinct A. fumigatus populations; specific alleles and genes involved in drug resistance were often distributed in a population-specific manner. Barber and collaborators (8) described the genomic variation associated with clinical isolates related to triazole resistance and virulence factors. Isolates were assigned to genetic clusters based on their single-nucleotide polymorphism (SNP) profiles and placed in the phylogeny, thus determining clusters enriched in clinical isolates, as well the functional genomic profiles of the isolates from the clusters (8). Another recent study determined the population structure of azole-resistant isolates from environmental and clinical sources, observing signatures of positive selection both in regions containing canonical genes encoding fungicide resistance in the ergosterol biosynthetic pathway and in regions that have no defined function (17).

Understanding the genetic divergence across isolates is essential for studying the genetic mechanisms underlying specific phenotypes, including differences in virulence and pathogenicity across A. fumigatus isolates. Here, we investigated if variation in ortholog gene content in populations of A. fumigatus isolates from different geographical regions is associated with isolate origin (clinical or environmental). To do so, we inferred orthologous groups of genes in the genomes of 206 globally distributed clinical and environmental isolates of A. fumigatus. Despite notable differences in orthogroup gene content across all isolates, no significant correlation between gene distribution and the origin of isolates was detected, pointing to similar orthogroup content between all clinical and environmental isolates when phylogeny is accounted for. These results support the hypothesis that clinical and environmental isolates of A. fumigatus do not differ in their gene content.

## RESULTS

### Orthology inferences among isolates.

**FIG 1:**
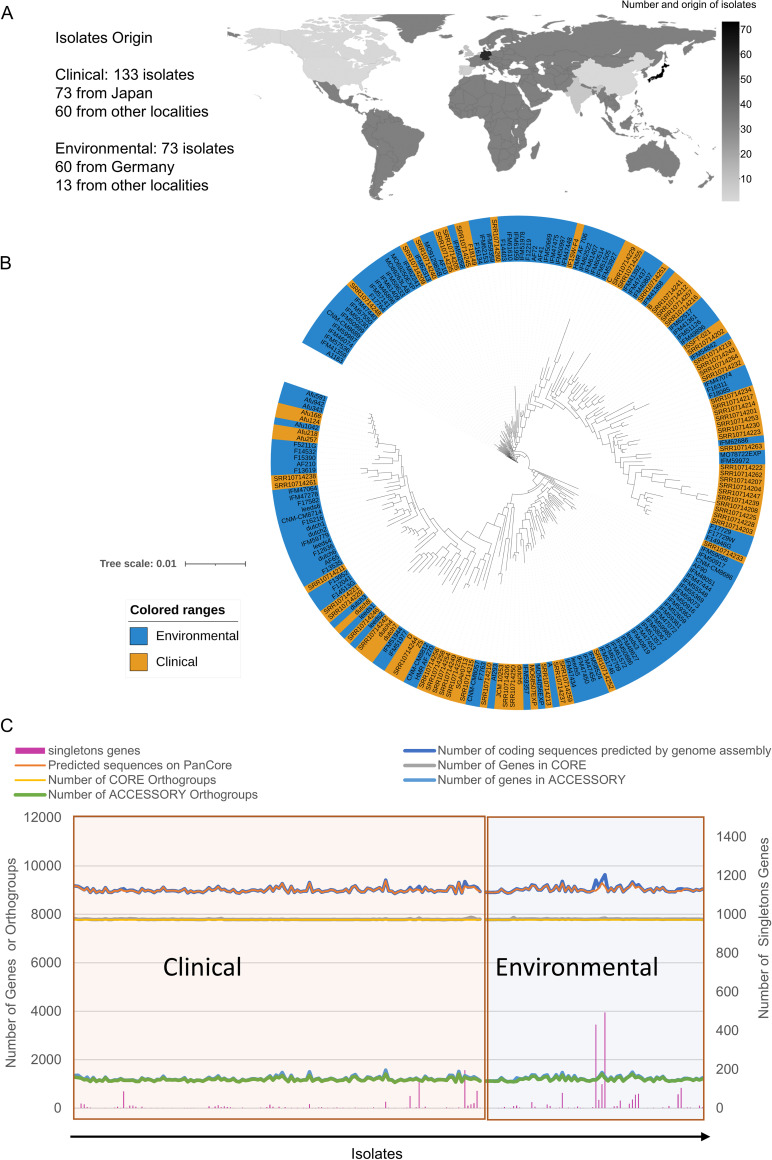
Isolate comparison by PanOrtho genome analysis, showing the high similarity of genomes in terms of phylogenetic distribution and gene classification, despite the worldwide distribution of isolates. (A) Geographical distribution of the isolates investigated showing the country origin. The darker coloration of countries represents an increase in the number of isolates. (B) Phylogenomic analysis of A. fumigatus isolates reveals that clinical and environmental isolates do not belong to distinct clades. The tree scale represents the evolutionary proximity represented by the clade’s distance. (C) Total numbers determined per isolate of clinical and environmental origin. The *x* axis shows isolates. The right *y* axis line plot represents the number of genes (genes classified in core and AC orthogroups per isolate, as well as the number of predicted genes for each isolate) or orthogroups (number of core and AC orthogroups present at each isolate); the left *y* axis bar plot represents the number of singleton genes determined in each isolate.

The examination of orthologous gene relationships among genes of all A. fumigatus genomes (genome metrics are presented in supplemental file 1 at https://figshare.com/articles/dataset/Examination_of_genome-wide_ortholog_variation_in_clinical_and_environmental_isolates_of_the_fungal_pathogen_Aspergillus_fumigatus/19873927) revealed a PanOrtho genome composed of core groups (i.e., genes present in all isolates), accessory (AC) groups (i.e., accessory genes present in two or more isolates), and isolate-specific orthogroups (referred to here as singletons). This genome represents 13,085 orthogroups, of which 7,773 (59.4% of the PanOrtho genome, corresponding to 1,606,449 genes) are core groups. AC groups plus singleton genes are represented in 5,312 groups (40.6% of the PanOrtho genome, corresponding to 252,316 genes), of which 2,656 are AC orthogroups with two or more genes (corresponding to 249,641 genes) (see supplemental file 2 at https://figshare.com/articles/dataset/Examination_of_genome-wide_ortholog_variation_in_clinical_and_environmental_isolates_of_the_fungal_pathogen_Aspergillus_fumigatus/19873927?file=36006890) and 2,675 orthogroups are composed by isolate specific genes, the singletons (see supplemental file 3 at the URL above). The number of orthogroups and predicted sequences classified for each isolate was also investigated to reveal the variation across isolates genomes and their origin (Fig. 1). Thus, we determined the frequency of isolates that share AC groups: 860 AC groups were identified in >95% of the isolates (6.6% of the PanOrtho genome), 774 groups were found in 5 to 95% of the isolates (5.9% of the PanOrtho genome), and 1,021 AC groups are present in fewer than 5% of the isolates (7.8% of the PanOrtho genome), showing the low variation in the distribution of orthologs across genomes (supplemental file 2). In addition, 2,675 genes isolate-specific, singletons represent 0.14% of the total number of genes of the data set and are exclusively found in 123 isolates (supplemental file 3). Further, we tested the default inflation parameter of Orthofinder for the PanOrtho construction (1.5). A total of 97.6% of the 4,132 conserved BUSCO families from the eurotiales_odb10 database were congruent (i.e., not fragmented) with our Orthofinder derived orthogroups; similar results were obtained with inflation parameters 2.0, 3.0, 4.0, and 5.0, indicating a minor effect of this parameter in our data set.

### Functional classification of PanOrtho genome.

The total number of genes in the PanOrtho genome was examined (see supplemental file 1 at https://figshare.com/articles/dataset/Examination_of_genome-wide_ortholog_variation_in_clinical_and_environmental_isolates_of_the_fungal_pathogen_Aspergillus_fumigatus/19873927) and compared with Af293 and A1163, the best-annotated and most commonly used reference isolates for A. fumigatus (18, 19). The average number of predicted genes for all genome was 9,023 ± 118; the genomes of clinical and environmental isolates had 9,008 ± 101 and 9,051 ± 140 genes, respectively. The frequency of core and AC groups was similar across isolates, with a slightly higher average number of genes for the environmental set of isolates (core: environmental, 9,027 ± 101, and clinical, 9,001 ± 93; AC: environmental, 1,223 ± 95, and clinical 1,205 ± 88). Notably, the average number of genes in the entire data set was nearly the same as the number of genes in the commonly used reference strains Af293 and A1163 (see supplemental Table 1 at the URL above). For the total data set, the average number of core genes was 9,010 ± 97, and that of AC genes was 1,211 ± 91 genes. To identify variation in genome content between isolates, the orthogroups were further classified using eggNOG v5.0 (20, 21) and confirmed by the function of orthogroup members from clinical reference isolates Af293 and A1163 (18, 19) (see supplemental file 4 at the URL above). For Af293, a total of 86.3% of the genes in its genome were core genes and 13.7% were AC genes, consistent with the predicted number of core genes across all genomes, corresponding to 86.4 ± 1.0% of genome size (see supplemental file 1).

Comparison of the ontology terms among genes present in core and AC orthogroups revealed differences in functional classifications (see supplemental Fig. 1 at https://figshare.com/articles/dataset/Examination_of_genome-wide_ortholog_variation_in_clinical_and_environmental_isolates_of_the_fungal_pathogen_Aspergillus_fumigatus/19873927). The “molecular adaptor activity” (GO:0060090) was present in the core group but absent in the AC group. The protein classification with the largest proportion in the core and AC group was “metabolite interconversion enzyme” (PC00262), and the protein class with the largest absence in AC group is “nucleic acid metabolism protein” (PC00171). In the AC group, there is a smaller proportion of peptidases and hydrolases acting on acid anhydrides (GO:0008233 and GO:0016817, respectively) than the core group; importantly, in the AC group, there is a larger proportion of hydrolases acting on glycosyl bonds and ester bonds (GO:0016798 and GO:0016788, respectively). Notably, higher relative proportions of oxidoreductase activity orthogroups were observed among the AC groups—specifically for “dioxygenase activity” (GO:0051213) and “oxidoreductase activity acting on peroxide as acceptor” (GO:0016684). These results demonstrate that core and AC groups of genes functionally differ.

**FIG 2:**
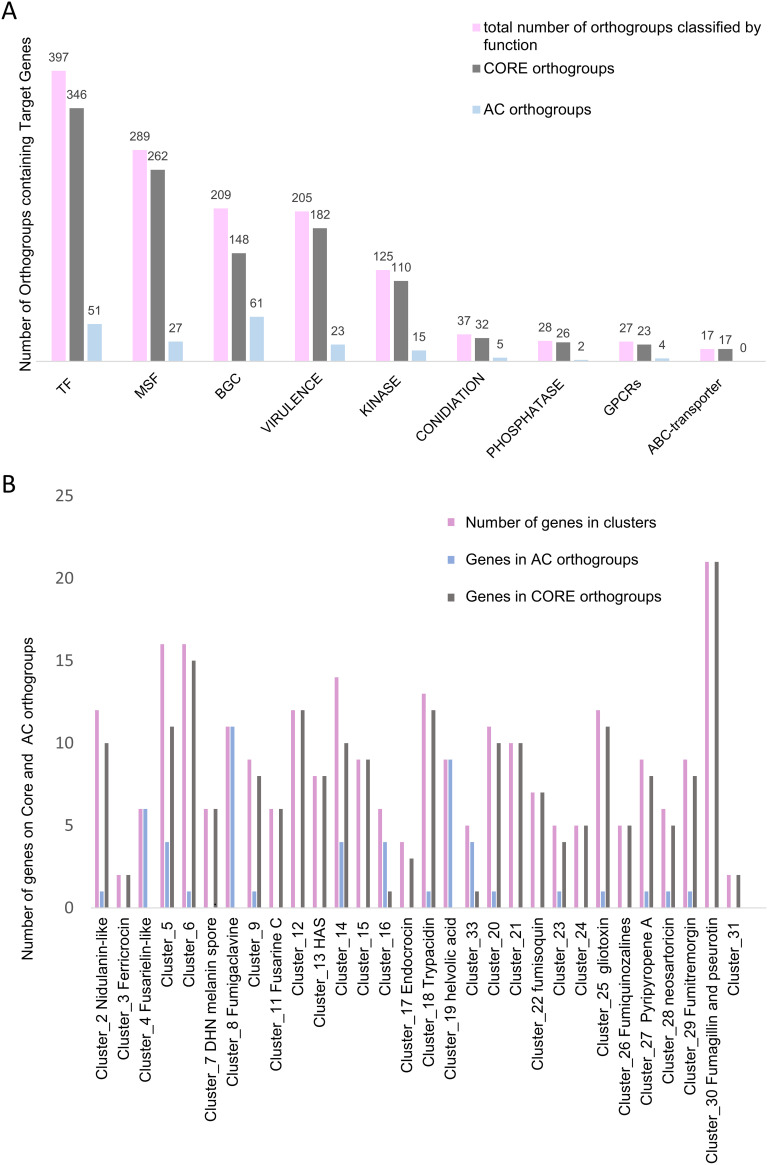
Analysis of target genes across A. fumigatus isolates shows that the variation in orthologous-gene distribution across isolates is a low-frequency event that usually affects fewer than 10 isolates (>5%), maintaining the majority of target genes classified on core orthogroups. (A) Genes putatively associated with A. fumigatus adaptability, survival, and virulence (or target genes) were localized in PanOrtho orthogroups and classified according to their function. (B) Gene presence-absence patterns among genes encoded in BGCs. TF, transcription factor; MFS, major facilitator superfamily transporter; BGC, biosynthetic gene clusters.

The annotation of the Af293 genome facilitated prediction of protein products encoded by 8,631 (66.0%) orthogroups (see supplemental file 4 at https://figshare.com/articles/dataset/Examination_of_genome-wide_ortholog_variation_in_clinical_and_environmental_isolates_of_the_fungal_pathogen_Aspergillus_fumigatus/19873927?file=36006890). Moreover, orthogroups putatively associated with A. fumigatus adaptability, survival, and virulence—which are referred to here as target genes (Fig. 2; also, see supplemental file 5 at the URL above)—could easily be identified. Target genes include kinase genes; conidiation-related genes; genes for G-protein-coupled receptors (GPCRs), phosphatases, ABC (ATP-binding cassette) transporters, major facilitator superfamily transporters (MFS), secondary metabolites (biosynthetic gene clusters [BGCs]), and transcription factors (TF); and genetic determinants of virulence. Across 1,334 orthogroups, we identified a total of 1,419 target genes, which spanned 188 AC and 1,146 core groups (Fig. 2A).

GPCR genes, conidiation genes, and phosphatase genes were observed among core groups. In contrast, genes associated with BGC, kinases, TF, and MFS transporters were observed among AC groups. For example, PkaC/Afu2g12200 (the catalytic subunit of cyclic AMP [cAMP]-dependent protein kinase) is present in a single copy across all isolates, whereas Fhk2/Afu3g07130 (a two-component response regulator) is present in a single copy in 101 isolates and present in multiple copies in 105 isolates (see supplemental file 5 at https://figshare.com/articles/dataset/Examination_of_genome-wide_ortholog_variation_in_clinical_and_environmental_isolates_of_the_fungal_pathogen_Aspergillus_fumigatus/19873927).

To determine variation among genes related to secondary metabolite production, we considered the biosynthetic gene cluster (BGC) classification from Lind et al. (6) that described the Af293 genetic clusters and the predicted functions for a partial number of clusters. For our analysis, we considered only those clusters where at least 80% of predicted genes were present as a BGC in each isolate consistent with the distribution of BGC as previously reported (6). According to this criterion, BGC 1, 10 and 33, which biosynthesize unknown secondary metabolites, are not conserved and were excluded (Fig. 2B; also, see supplemental file 5 at https://figshare.com/articles/dataset/Examination_of_genome-wide_ortholog_variation_in_clinical_and_environmental_isolates_of_the_fungal_pathogen_Aspergillus_fumigatus/19873927). A total of 270 genes from BGC were identified on the orthogroups, of which 81.4% are in core and 18.5% are in AC groups. The gliotoxin BGC is conserved, 12 of 13 genes are core genes, and only isolate IFM59779 lacks genes orthologous to *gliK* (Afu6g09700). To further support this finding, we mapped the IFM59779 reads onto the Af293 genome and found that reads did not map to *gliK* supportive of gene loss (see supplemental Fig. 2 at the URL above). The same conservation patterns were observed for helvolic acid (cluster 19), and nine genes were identified among core and AC groups, respectively. Of note, the helvolic BGC was absent in CNM-CM8812, suggestive of an isolate-specific loss of genomic content.

### Gene copy number variation.

Another important aspect that may contribute to organismal adaptive evolution and the evolution of fungal pathogens is the variation on gene copy number (22–24). To evaluate gene copy number variation, we examined the number of genes assigned for each isolate per orthogroup. Multicopy genes were observed at a low frequency across isolates. Specifically, in 8.4% of the PanOrtho genome (1,094 groups, 478 AC and 616 core) there was at least one isolate with multiple gene copies and in 1.1% (146 orthogroups, 78 AC and 68 core) of the PanOrtho genome 5% of isolates (i.e., more than 10 isolates) had two gene copies. Orthogroups with isolates that encoded three gene copies were observed in 1.5% of the PanOrtho genome (194 groups, 133 AC and 61 core). In only 14 groups (0.1% of the PanOrtho genome, 13 AC and 1 core), three gene copies were observed in 5 to 23% of isolates (see supplemental file 2 at https://figshare.com/articles/dataset/Examination_of_genome-wide_ortholog_variation_in_clinical_and_environmental_isolates_of_the_fungal_pathogen_Aspergillus_fumigatus/19873927?file=36006890).

**FIG 3:**
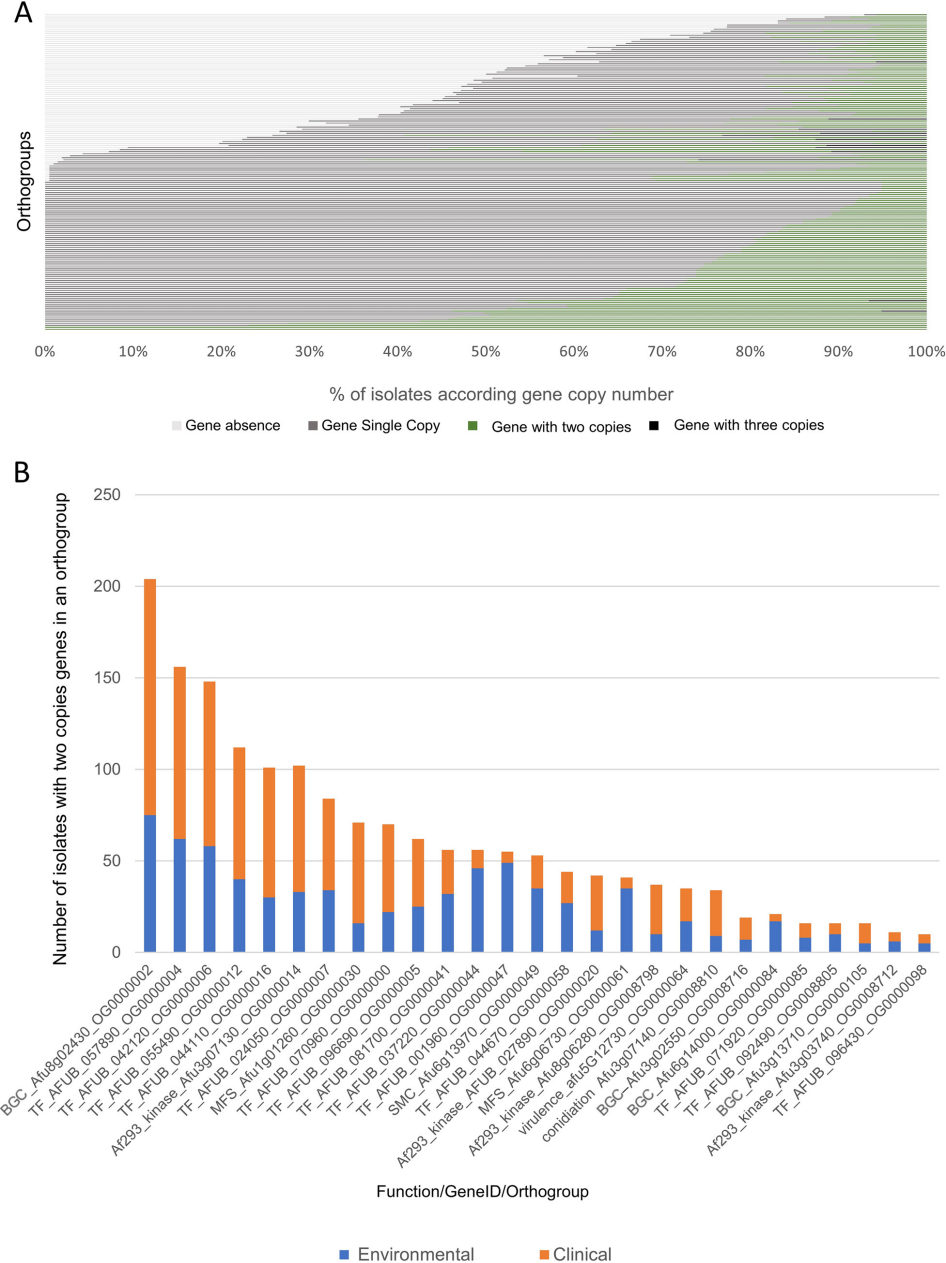
The copy number variation detected in PanOrtho genome shows differences in the proportion of absent and multiple-copy genes across orthogroups and in the distribution of the isolates with two-copy genes. (A) Distribution of isolate presence and absence per orthogroup. Genes present in a single copy are shown in gray, genes present in two copies are in green, genes present in three copies are in dark gray, and genes that are absent are in light gray. (B) Target genes present in two copies and their differences in the distribution of isolates within orthogroups according to clinical or environmental origin.

We next evaluated gene copy number variation among orthogroups presenting multicopy genes in more than 5% of isolates (Fig. 3A; also, see supplemental file 2 at https://figshare.com/articles/dataset/Examination_of_genome-wide_ortholog_variation_in_clinical_and_environmental_isolates_of_the_fungal_pathogen_Aspergillus_fumigatus/19873927?file=36006890). In addition, we detailed the variation of two copies genes according to isolates origin for the target genes, genes putatively associated with A. fumigatus adaptability, survival, and virulence (Fig. 3B; also, see supplemental file 5 at the URL above). Genes present in two or three copies were observed among 14 orthogroups with varying frequency across isolates—more specifically, 9.7% to 42% of isolates had two genes present in an orthogroup, and 5.3% to 23% had three genes present in an orthogroup. Among these 14 orthogroups, OG0000013, which encodes a putative amino oxidase, is present in the core group. The remaining 13 are observed in the AC group and include genes of diverse functional categories and domains: no protein function classification (unknown function DUF3435, conserved hypothetical protein) or predicted FAD-binding domain, transposition, RNA-mediated, and Chromo domain protein function. Notably, the orthogroup OG0000009, which was observed in the AC group, has a putative protein function related to the constitution of regulatory subunit 26S proteasome, the major nonlysosomal protease in eukaryotic cells. This multiprotein complex is involved in the ATP-dependent degradation of ubiquitinated proteins, playing a key role in the maintenance of protein homeostasis by removing misfolded or damaged proteins (25). Among all isolates, 49% had two gene copies (39% environmental and 61% clinical isolates) and 5% had three copies (89% environmental and 11% clinical isolates) of OG0000009. Two gene copies were also observed in orthogroups carrying TF genes—for example, 27.2% of isolates had two copies assigned to orthogroups OG0000044 (Forkhead transcription factor Fkh1/2) and OG0000047 (C6 finger domain protein). Of note, the distribution of isolates by origin in both orthogroups is different from the distribution across the whole data set (35.5% environmental and 64.5% clinical isolates across all orthogroups). Specifically, in OG0000044 and OG0000047, two gene copies were present in 82% and 87.5%, respectively, among environmental isolates, whereas two gene copies were observed among 18% and 12.5% of clinical isolates (supplemental file 2 [URL above]).

Zinc finger transcription factors often control the expression of multiple genes and trigger control cascades that dramatically influence the regulation of abiotic stresses (26), including influencing cation homeostasis and the detoxification process in Aspergillus nidulans (27). Two orthogroups that encode zinc finger TF had high frequencies of isolates presenting two copies of the genes —specifically, 72.8% and 50.5% of isolates, respectively, on orthogroups OG0000006 and OG0000016 (see supplemental file 2 at https://figshare.com/articles/dataset/Examination_of_genome-wide_ortholog_variation_in_clinical_and_environmental_isolates_of_the_fungal_pathogen_Aspergillus_fumigatus/19873927?file=36006890). Specifically, 37.3% and 62.7% of environmental and clinical isolates, respectively, had two gene copies present in orthogroup OG0000006; 28.8% and 71.1% of environmental and clinical isolates, respectively, had two gene copies present in orthogroup OG0000016. As the frequency expected for the whole data set according to the isolates’ origin is 35.5% environmental and 64.5% clinical, we observed an increase in copy number only for OG0000016, which presented variation above 5% compared to the data set normal distribution.

Another group of genes often present in multiple copies is those encoding signal sensor histidine kinases. For orthogroups OG0000012 and OG0000014, 55.8% and 48.5% of isolates had two gene copies in each orthogroup, respectively (see supplemental file 2 at https://figshare.com/articles/dataset/Examination_of_genome-wide_ortholog_variation_in_clinical_and_environmental_isolates_of_the_fungal_pathogen_Aspergillus_fumigatus/19873927). In the opportunistic human-pathogenic yeast Candida albicans, these proteins encode two-component signaling proteins that transmit signals through high-osmolarity glycerol response 1 (HOG1) and mitogen-activated protein kinase (MAPK cascade) in response to environmental osmotic stimuli (28, 29). In A. fumigatus, HOG and cell wall integrity pathways are important for the adaptation to different forms of environmental adversity such as osmotic and oxidative stresses, nutrient limitations, high temperatures, and other chemical and mechanical stimuli (30–32). The frequency of two gene copies with respect to isolate origin was similar to the frequencies of environmental (35.5%) and clinical (64.5%) isolates across the whole data set, suggesting no differentiation of multiple copies according to origin. Overall, the majority of the orthogroups (68.7%; 8,997 orthogroups) of the PanOrtho genome had a similar distribution of isolate origin of the data set (64.5% clinical and 35.5% environmental), considering a standard deviation of 5% for isolate distribution.

We next tested whether increased copy number was observed among target genes, in which multiple-copy genes detected in more than 5% of isolates are in orthogroups that encode TF, BGC, and kinases, respectively, i.e., 12, 8, and 5 orthogroups (Fig. 3B; also, see supplemental file 5 at https://figshare.com/articles/dataset/Examination_of_genome-wide_ortholog_variation_in_clinical_and_environmental_isolates_of_the_fungal_pathogen_Aspergillus_fumigatus/19873927).

To determine if gene copy number per orthogroup varied between environmental and clinical isolates, we conducted a phylogenetic analysis of variance (phylANOVA) (33). To do so, single-copy orthologs were first used to infer the evolutionary history of the 206 isolates (Fig. 1A and B; unrooted tree provided in supplemental Fig. 3 at https://figshare.com/articles/dataset/Examination_of_genome-wide_ortholog_variation_in_clinical_and_environmental_isolates_of_the_fungal_pathogen_Aspergillus_fumigatus/19873927). In these, we did not observe a significant number of associations between the phylogenetic position of an isolate and its status of clinical or environmental origin (*P* > 0.05; phylANOVA). These findings suggest that when evolutionary history is accounted for, environmental and clinical isolates do not substantially differ in gene copy number per orthogroup. Consistent with this observation, the number of classified sequences on the PanOrtho genome and the orthogroups classified for each isolate maintain a linear trend across clinical and environmental isolates (Fig. 1C).

### Origin of singletons.

To investigate the possible origin of the orthogroups of singletons genes, each sequence was searched against all fungal and bacterial genes from the nonredundant protein database (nr-NCBI) using BLAST (34). The “Sugar transporter (major facilitator superfamily [TC 2.A.1.1])” and “Fungal specific TF domain” were the functional descriptions most frequently observed as best BLAST hits. For the most part, the singleton genes contain sequences with orthologs in fungi and sequences with a high degree of differentiation, with orthologs in a different genus or no ortholog at all (see supplemental file 3 at https://figshare.com/articles/dataset/Examination_of_genome-wide_ortholog_variation_in_clinical_and_environmental_isolates_of_the_fungal_pathogen_Aspergillus_fumigatus/19873927).

### Transposable elements.

Mobilization of transposons can cause a variety of localized effects on genomes, such as gene inactivation or modification, and they are often found in multiple, highly conserved copies. Transposons also represent potential hot spots for the occurrence of inversions, translocations, deletions, and gene copies (35). In A. fumigatus, two nonfunctional type I transposons (AfutI and AfutII) (36, 37), a seemingly nonfunctional class II element (Taf1) (38), and a type II transposon of the Tc1/Mariner superfamily (Aft1) (39) have been reported. The search for transposons Aft1 and Taf1 in all A. fumigatus genomes was performed considering nucleotide identity (see supplemental file 6 at https://figshare.com/articles/dataset/Examination_of_genome-wide_ortholog_variation_in_clinical_and_environmental_isolates_of_the_fungal_pathogen_Aspergillus_fumigatus/19873927). This analysis revealed variation in the number of repeated elements across all analyzed genomes (see supplemental Fig. 4 at the URL above). It has been reported that Taf1 is present in different locations and copy numbers among clinical isolates of A. fumigatus (38, 39). We identified 20 copies of Aft1 (EF100757.1; 1,882 bp) and 13 copies of Taf1 (AY971670.1; 2,023-bp sequence) with nucleotide identity of >99.0% in the isolate Af293 (see supplemental file 6 at the URL above). Aft1 was found in 190 isolates in 1 to 20 copies, and Taf1 was present in 204 isolates, at copy numbers varying from 1 to 18 (see supplemental Fig. 4 and supplemental file 6). The isolate with the highest number of copies for both elements was the environmental strain SGAir0713 (18 copies of Taf1; 21 copies of Aft1). TAf1 orthologs were not identified in the environmental isolates B-1-26-5 and C-1-27s-1. According to the findings from previous studies (35, 40), we hypothesize that transposable element variation may be associated with the observed phenotypic differences reported.

## DISCUSSION

Aspergillus taxonomic sections show extensive variation, and the traits and genetic elements that contribute to this variation are still under investigation (41–43). Previous comparative genomic investigations in the genus Aspergillus characterize both species diversification and variation within species (44). Aspergillus fumigatus is an opportunistic pathogen, and the traits (if any) that allow an environmental isolate to cause clinical infection are still unclear. The phenotypic heterogeneity of the specie is related to what has been observed either in clinical or environmental isolates. Unfortunately, most of the current isolates are not physically available to us, making their phenotypic characterization impossible. The first study that investigated the distribution of orthologous genes in A. fumigatus used 12 isolates and identified 8,073 core groups and 1,964 AC groups (16). The increasing availability of sequencing data allowed even more precise descriptions of the A. fumigatus pancore genomes, and notably, the genome dataset described here partially overlaps the most recently described pangenome studies (8, 10), mainly due to the A. fumigatus genomes available in the NCBI database, which are of extreme importance as reference strains for A. fumigatus studies, such as Af293, A1163, CEA17, and others. The largest pancore genome analysis among 300 A. fumigatus isolates (83 clinical and 217 environmental, the majority being from Germany) revealed that the A. fumigatus pangenome is larger than previously estimated, suggesting the relatively closed pangenome structure of species when considering only AC gene families (8). Corroborating these findings, we observed small variation across genes classified in core and AC orthogroups, as well as across total predicted genes for each isolate (Fig. 1C). We identified numbers of total orthogroups in core and AC sets comparable to that reported by Barber and collaborators (8), which may be in part due to partial overlap between isolates used in both studies; however, we note that our dataset contained approximately twice the number of clinical isolates. Both studies report infrequent variation, with fewer than 5% of the isolates among genes related to virulence (Fig. 2; also, see supplemental file 2 at https://figshare.com/articles/dataset/Examination_of_genome-wide_ortholog_variation_in_clinical_and_environmental_isolates_of_the_fungal_pathogen_Aspergillus_fumigatus/19873927) (8). Nevertheless, the distribution of orthologs cannot be directly compared between the studies, because the partial overlap between data sets predicts different results for the ortholog gene classification. While we investigated the distribution of the ortholog genes in orthogroups to determine the influence of orthologous distribution on the phylogenetic inference of clinical and environmental isolates, Barber et al. investigated infrequent variation associated with clinical isolates and triazole resistance, as well characterize genetic variation in known virulence factors. We believe that the two works used the orthologs’ intraspecies analysis to deeply investigate different aspects of A. fumigatus genome variation. Thus, the distribution of orthologous genes provided here contains information important to the Aspergillus research community (see supplemental file 2 at the URL above), as well the possibility of identifying Af293 ortholog genes on the PanOrtho genome (see supplemental file 4 at the URL above).

Across all isolates, we found that A. fumigatus harbors variation in terms of the number of total predicted protein coding genes ranging between 8,857 and 9,638 (for the clinical isolate IFM59779 and environmental isolate SRR10714233/B-1-26-5, respectively) (see supplemental Table 1 at https://figshare.com/articles/dataset/Examination_of_genome-wide_ortholog_variation_in_clinical_and_environmental_isolates_of_the_fungal_pathogen_Aspergillus_fumigatus/19873927). The isolates with the largest number of genes classified exclusively as core genome were the clinical isolate MO78722EXP and the environmental isolate ISSFT-021, which was obtained from the International Space Station, with 7,877 and 7,867 genes classified, respectively. In the AC set, the clinical isolates IFM59361 and 12-7504462 had the highest number of AC classified genes, 1,570 and 1,521, respectively (see supplemental file 7 at the URL above), revealing variation in gene copy numbers (no, single, or multiple gene copies) across isolates (Fig. 3A). Other studies suggest that the genetic variants—SNPs, indels, and gene presence-absence polymorphisms—across A. fumigatus isolates may provide evidence of distinct populations of A. fumigatus (10, 17).

We investigated the distribution of genes with functional associations with relevant pathways investigated in A. fumigatus studies in the PanOrtho genome, focusing on important mechanisms significant to the phenotypic differentiation of isolates (see supplemental file 5 at https://figshare.com/articles/dataset/Examination_of_genome-wide_ortholog_variation_in_clinical_and_environmental_isolates_of_the_fungal_pathogen_Aspergillus_fumigatus/19873927). Our analysis revealed extensive variation in copy number among genes encoding GPCRs, phosphatases, ABC transporters, kinases, TF, MSF transporters, and proteins important for conidiation, virulence, and secondary-metabolite production (Fig. 2A). Notwithstanding these differences, gene copy number per orthogroup did not differ between clinical and environmental isolates.

A. fumigatus produces a variety of secondary metabolites (SM) and efflux pumps that serve as defense systems (45, 46). In fungi, the genes in pathways that synthesize SM are typically located next to each other in the genome and organized in contiguous gene clusters (BGC) (6, 47–49). The gliotoxin BGC impacts A. fumigatus virulence and is widely produced by Aspergillus species (50, 51). Here, we observed the conservation of the gliotoxin BGC across the PanOrtho genome (Fig. 2B). However, other BGCs, such as fumitremorgin, presented heterogeneity with regard to the genetic arrangement of BGCs within species (50), an observation that is typified by the total absence of these genes in the environmental isolate B-1-70s-1 (see supplemental file 5 at https://figshare.com/articles/dataset/Examination_of_genome-wide_ortholog_variation_in_clinical_and_environmental_isolates_of_the_fungal_pathogen_Aspergillus_fumigatus/19873927). Of the genes belonging to BGCs, 27 presented significant variation in species distribution in the PanOrtho genome (Fig. 2B). All genes from the helvolic acid BGC were absent from isolate CNM-CM8812. All genes in BGC 4, predicted to produce a fusarielin-like metabolite, were classified as accessories and are absent in different isolates, such as A1163 (ASM15014v1), which lost all genes from this BGC. The fumitremorgin BGC, cluster 29, is present in a unique isolate (Afu343) with two copies of all genes of the BGC. Of note, our analysis examined the presence and absence of genes encoded in BGCs, but further examination of physical clustering is warranted. Nonetheless, our findings corroborated previous descriptions of variation among BGCs within this species (6–8).

The genetic diversity across species in virulence and drug resistance mechanisms has been extensively reviewed (43, 46, 52). Among clinical isolates of Aspergillus species, species- and isolate-specific polymorphisms were reported in the 14α-sterol demethylase gene *cyp51A* (Afu4g06890) and in the 1,3-beta-glucan synthase catalytic subunit gene *fks1*, which are target genes for azoles and echinocandins, respectively (7, 14, 42). We observed no variation in gene copy number for *cyp51A* (Afu4g06890; orthogroup OG0003434), *cyp51B* (Afu7g03740; orthogroup OG0000331), and *fks1* (Afu6G12400; orthogroup OG0003140); these were all members of core groups (see supplemental files 2 and 4 at https://figshare.com/articles/dataset/Examination_of_genome-wide_ortholog_variation_in_clinical_and_environmental_isolates_of_the_fungal_pathogen_Aspergillus_fumigatus/19873927).

The genetic determinants of A. fumigatus virulence were previously described (51) and appear as a conserved set of genes on our PanOrtho genome. Of 204 genes studied, only 23 were classified in AC groups. The gene *pes3*, encoding nonribosomal peptide synthetase 8 (Afu5G12730), is localized in the core orthogroup OG0000064 and is present in two copies for 38 isolates (44.7% environmental and 55.2% clinical). The AC group OG0008770 contains the important virulence factor gene *cgrA* (Afu8G02750; nucleolar rRNA processor) and shows strong variation: 50 isolates do not contain the gene (80% environmental and 20% clinical), and 156 isolates have a single copy of the gene (20.5% environmental and 79.5% clinical), suggesting a tendency to lose this gene in environmental isolates. The variation in AC is mainly due to the absence of orthologs in one or two isolates, and CNM-CM8812 lacked orthologous genes in nine orthogroups encoding genetic determinants of virulence.

Conidiation is critical for A. fumigatus dispersal to new environments, including the human lung (53). FluG (Afu3g07140; OG0008810) acts upstream of the BrlA activator of conidiation (54, 55) and was absent in 96 isolates (41.7% environmental and 55.2% clinical), present in a single copy in 76 isolates (30.6% environmental and 69.7% clinical), and present in two copies in 34 isolates (26.5% environmental and 73.5% clinical). The extensive absence of *FluG* orthologs suggests that FluG may not be essential for conidiation.

In environmental isolates, the impact of fungicides on the number of gene duplications in A. fumigatus is variable and the overall resistance frequency among agricultural isolates is low (1, 15) relative to the increasing azole resistance trend in clinical A. fumigatus isolates (56). Isolates collected from soil after the growing season and azole exposure show a subtle but consistent decrease in susceptibility to medical and agricultural azoles (15). Whole-genome sequencing indicates that despite variation in antifungal susceptibility, fungicide application does not significantly affect the population structure and genetic diversity of A. fumigatus in the fields (15), consistent with our finding of conservation in orthogroup distribution among clinical and environmental isolates.

PanOrtho genome analysis allowed us to detect instances of copy number variation, especially when multiple gene copies were present. For example, among 56 isolates that carry two copies of the TF-encoding genes Afu1g01560 (OG0000047) and Afu3g11960 (OG0000044), 87.5% and 82%, respectively, are environmental isolates whereas 12.5% and 18%, respectively, are clinical isolates. Two genes encoding kinase activity—Afu8g06280 (OG0008798) and Afu3g07130 (OG0000014)—tended to be more widely observed among clinical isolates; 73.6% and 69% were clinical isolates harboring two copies, whereas 26.3% and 31% were environmental isolates, respectively. The observed variation qualitatively differed from the variation observed across all orthogroups (35.5% environmental and 64.5% clinical). We also highlight transposable elements *Taf1* and *Aft1* as a source of substantial genetic variation among A. fumigatus isolates.

Our work investigated variation in orthogroup content among a comprehensive panel of clinical and environmental isolates of A. fumigatus. Despite the identification of substantial variation in gene presence, absence, and copy number across the whole dataset, no variation was observed among environmental and clinical isolates when accounting for phylogeny. This finding raises the hypothesis that environmental and clinical isolates may differ due to other types of genetic variation (e.g., SNPs and indels) or do not substantially differ at all.

## MATERIALS AND METHODS

### Dataset assembly.

The data set was constructed based on previously determined genomes (15, 50, 57). The isolate descriptions are provided in supplemental file 7 at https://figshare.com/articles/dataset/Examination_of_genome-wide_ortholog_variation_in_clinical_and_environmental_isolates_of_the_fungal_pathogen_Aspergillus_fumigatus/19873927. Three principal data sets were used (details are in supplemental file 1 at the URL above). (i) Clinical isolates from different Japanese cities were provided through the National Bio-Resource Project (NBRP), Japan (http://nbrp.jp/) (NCBI BioProject no. PRJNA638646), and additional isolates (58) from NCBI BioProject no. PRJDB1541 originated from different patients, sources, and infections, described by Zhao et al. (57, 59). (ii) Public genomes available from clinical and environmental isolates were used, including Af293 (GenBank accession no. GCA_000002655.1), CEA10 (strain synonym, CBS 144.89/FGSC A1163; GenBank accession no. GCA_000150145.1), HMR AF 270 (GenBank accession no. GCA_002234955.1), and Z5 (GenBank accession no. GCA_001029325.1), and additional isolates from Spain (42), Portugal, and other origins were assembled (50, 60) and made public under NCBI BioProject PRJNA577646. (iii) Environmental soil samples from German fields before and after fungicide application were used to isolate A. fumigatus isolates. The DNA extraction, genome sequencing, and quality assessment are described in reference 15, and the reads under BioProject no. PRJNA595552 were trimmed using Trimmomatic v0.36 (61) and used as input in SPAdes v3.11.1 (63) to obtain the genome assemblies. The assemblies from studies 2 and 3 were kindly provided by the groups of A. Rokas and J. G. Gibbons. The samples originated from 133 clinical and 73 environmental isolates. Clinical isolates are largely from Japan (73 isolates) and Europe (55 isolates), and environmental isolates are from German (60 isolates) soil samples (15) and other sources (see supplemental file 7).

### Data analysis.

All genome assemblies were tested by quality metrics and scaffold size evaluation through QUAST (82) (see supplemental file 1 at https://figshare.com/articles/dataset/Examination_of_genome-wide_ortholog_variation_in_clinical_and_environmental_isolates_of_the_fungal_pathogen_Aspergillus_fumigatus/19873927). A new A. fumigatus training model was constructed and used during the consensus prediction sequences process by the software AUGUSTUS, v3.3.2 (65), for all assemblies. To determine gene content completeness, we used the BUSCO v2.0.1 pipeline (64) to examine assembly contigs for the presence of nearly universally single-copy orthologs (see supplemental Fig. 5 at the URL above). The *Eurotiales* OrthoDBv10 data set (66) of nearly universally single-copy orthologs was used. Our analyses identified genomes with a maximum of 20 missing BUSCO genes. These metrics suggest that all 206 A. fumigatus genomes are nonfragmented and suitable for comparative genomic analyses. Genome metrics are presented in supplemental file 1, and the BUSCO classification for isolate genomes is presented in supplemental Fig. 5.

To identify gene families across genomes, we used a Markov clustering approach. Specifically, we used OrthoFinder v2.3.8 (67), with an inflation parameter of 1.5. After normalizing BLAST bit scores, genes were clustered into discrete orthogroups using a Markov clustering approach (68). The eggNOG-mapper was used to assign GO, KEGG, and Pfam terms to orthogroups based on their orthology relationships (20, 21). For that purpose, we selected the eukaryotic eggNOG database (euNOG). Protein-coding genes were further classified by Gene Ontology terms using Panther v16 (69).

### Matrix construction and statistical analysis.

Based on OrthoFinder results, two matrices were constructed, one that described the genes in the orthogroups and one that describes the number of genes from each isolate in the orthogroups. The gene-counting matrix established the number of absent, single-copy, and multiple-copy genes per isolate (see supplemental file 2 at https://figshare.com/articles/dataset/Examination_of_genome-wide_ortholog_variation_in_clinical_and_environmental_isolates_of_the_fungal_pathogen_Aspergillus_fumigatus/19873927). Based on the gene identification matrix, the gene IDs of target orthogroups were selected to obtain the respective coding sequences. The set of coding sequences was further annotated using eggNOG 5.0 (21), and the functional profile was constructed based on the annotation of the sequence. The statistical computing and data analysis were done using R v4.1.2 (70). We considered orthogroups with variation in more than 5% of isolates to avoid orthogroups with false-positive cases due to assembly issues.

To reconstruct the evolutionary history of all A. fumigatus isolates, we implemented a workflow similar to previously established protocols (71). More specifically, we first individually aligned amino acid sequences of single-copy orthologs using MAFFT v7.402 (72) with the “auto” mode. Next, codon-based alignments were generated by threading the corresponding nucleotide sequences onto the amino acid alignments using the “thread_dna” function in PhyKIT v1.2.1 (73). The resulting codon-based alignments were trimmed using ClipKIT v1.1.5 (74) with the “smart-gap” mode. To save computational time during strain tree inference, we subsampled all single-copy orthologs for the 2,500 most phylogenetically informative according to the number of parsimony-informative sites in each alignment. The number of parsimony-informative sites in each alignment was calculated using the “pis” function in PhyKIT. The 2,500 most informative single-copy orthologs were concatenated into a single supermatrix using the “create_concat” function in PhyKIT. The resulting alignment had 3,937,461 total sites, 70,307 parsimony-informative sites, and 3,799,815 constant sites, which was determined using the “aln_summary” function in BioKIT v0.0.5 (75) and used as input into IQ-TREE v2.0.6 (75, 76), generating tree 1. The best-fitting substitution model, GTF+F+I+G4 (77, 78), was determined using the Bayesian information criterion in IQ-TREE. Bipartition support was assessed using 5,000 ultrafast bootstrap approximations (79). phylANOVA (33, 80, 81) was used to compare gene copy number per orthogroup among environmental and clinical isolates, using a *P*-value threshold of 0.05. The *P*-value results are described for each orthogroup in supplemental file 2 (https://figshare.com/articles/dataset/Examination_of_genome-wide_ortholog_variation_in_clinical_and_environmental_isolates_of_the_fungal_pathogen_Aspergillus_fumigatus/19873927). To determine if the observed phylANOVA results were sensitive to the inferred genome-scale phylogeny, we inferred a second phylogeny using parsimony-informative sites from the 5,000 genes with the most parsimony-informative sites. The concatenation matrix was constructed by creating a supermatrix from the 5,000 genes, and parsimony-informative sites were retained using the “kpi” mode of trimming in ClipKIT v1.1.5 (74). The resulting concatenation matrix (93,176 sites) was used to reinfer the phylogeny using the same parameters as before, generating phylogenetic tree 2.
